# Optimization Strategies of Hybrid Lithium Titanate Oxide/Carbon Anodes for Lithium-Ion Batteries

**DOI:** 10.3390/nano14221799

**Published:** 2024-11-09

**Authors:** Maria Apostolopoulou, Dimitra Vernardou, Stefano Passerini

**Affiliations:** 1 Department of Electrical and Computer Engineering, School of Engineering, Hellenic Mediterranean University, 71410 Heraklion, Greece; mapost@hmu.gr; 2 Helmholtz Institute Ulm, Karlsruhe Institute of Technology, Helmholtzstrasse 11, 89081 Ulm, Germany; stefano.passerini@kit.edu; 3 Austrian Institute of Technology, Transport Technologies Giefinggasse 4, 1210 Wien, Austria

**Keywords:** lithium-ion batteries, lithium titanate oxide, synthesis methods, hybrid Li_4_Ti_5_O_12_/carbon-based materials

## Abstract

Lithium-ion batteries, due to their high energy density, compact size, long lifetime, and low environmental impact, have achieved a dominant position in everyday life. These attributes have made them the preferred choice for powering portable devices such as laptops and smartphones, power tools, and electric vehicles. As technology advances rapidly, the demand for even more efficient energy storage devices continues to rise. In lithium-ion batteries, anodes play a crucial role, with lithium titanate oxide standing out as a highly promising material. This anode is favored for its exceptional cycle stability, safety features, and fast charging capabilities. The impressive cycle stability of lithium titanate oxide is largely due to its zero-strain nature, meaning it undergoes minimal volume changes during lithium-ion insertion and extraction. This stability enhances the anode’s durability, leading to longer battery life. In addition, the lithium titanate oxide anode operates at a voltage of approximately 1.55 V vs. Li^+^/Li, significantly reducing the risk of dendrite formation, a major safety concern that can cause short circuits and fires. The material’s spinel structure, with its large active surface area, further allows fast electron transfer and ion diffusion, facilitating fast charging. This review explores the characteristics of lithium titanate oxide, the various synthesis methods employed, and its integration with carbon materials to enhance cycle stability, coulombic efficiency, and safety. It also proposes strategies for optimizing lithium titanate oxide properties to create sustainable anodes with reduced environmental impact using eco-friendly routes.

## 1. Introduction

In recent years, energy storage devices have become essential across various applications, from portable electronics to electric vehicles. The demand for high-performance rechargeable lithium-ion batteries (LIBs) has increased dramatically, leading to numerous efforts to develop advanced electrically conductive materials. Among the critical components of LIBs, the anode plays a pivotal role as the primary site for lithiumions during charging and discharging. The choice of anode material not only influences the overall capacity and energy density of the battery but also affects its charge rate, cycle life, and safety features [1].

Lithium titanate oxide (LTO) is considered an ideal anode material for LIBs due to its excellent coulombic efficiency (almost 100%), theoretical capacity of ~175 mAhg^−1^ [2], stable discharge voltage, and compatibility with a range of electrolytes. These include organic electrolytes such as LiPF_6_ dissolved in different ratios of organic solvents including ethylene carbonate (EC), propylene carbonate (PC), dimethyl carbonate (DMC), diethyl carbonate (DEC), and ethylene carbonate (EMC) [3,4], as well as aqueous electrolytes such as LiCl and Li_2_SO_4_ in water [1,5]. Additionally, LTO is non-toxic and relatively inexpensive and performs well at low temperatures, showing excellent reversibility in lithium-ion reactions. Its minimal volume change during lithium-ion insertion/extraction ensures excellent cycle stability and minimizes the risk of dendrite formation—a common issue with carbon-based anodes [1]. Furthermore, LTO’s high lithium insertion potential of approximately 1.55 V compared to Li/Li^+^ lies outside the electrochemical stability window of typical organic electrolytes, preventing lithium metal plating and enhancing safety [1].

However, a major issue with the LTO anode is the production of gases such as H_2_, CO_2_, and CO. These gases result from reactions between the anode interface and the organic solvents in the electrolyte, particularly on the (111) plane of the LTO crystalline lattice. This reaction changes the atomic orientation to (222) and then to (101), forming a TiO_2_ anatase structure that releases H_2_ gas, resulting in electrode deterioration [2,4]. Additionally, LTO’s initial electronic conductivity and diffusion coefficient of 10^−8^~10^−15^ cm^2^s^−1^ are relatively low, largely due to its structural characteristics and the reactions that alter its crystal structure [1]. To address these issues, researchers are exploring strategies such as the donor doping of the LTO crystal lattice(i.e., using high-valence elements to replace Li (Mg^2+^, Al^3+^, La^3+^, Ca^2+^, Zn^2+^, Sn^4+^), Ti (Nb^5+^, W^6+^,V^5+^, Mo^6+^, Ta^5+^), or O^2−^ (F^−^, Br^−^)), which can provide mixed-valence Ti^3+^/Ti^4+^ as charge compensation and thereby create n-type electronic conductivity in Li_4_Ti_5_O_12_ [1]. Another strategy is surface treatment (i.e., carbon coating to optimize electrical conductivity and chemical stability, nanoparticles of metals such as gold to promote the kinetics of the electrode reaction, heat treatment) [1]. All the above contribute to reducing gas generation and improving the ionic diffusion of the LTO anode. Another parameter that affects the performance of LTO electrodes is temperature. At low temperatures, the electrochemical performance decreases due to increased electrolyte viscosity and reduced ionic conductivity, which slows down the movement of lithium ions. For example, at −40 °C, LTO electrodes retain only about 55% of their capacity at room temperature, according to the literature. In contrast, high temperatures (e.g., 55 °C) increase the internal resistance due to the thicker SEI layer formed, resulting in a loss of capacitance [6,7]. These findings suggest that LTO anodes operate most effectively at room temperature. Moreover, when evaluating the effect of temperature on the stability of LTO during continuous cycling, it is worth mentioning the excellent cyclic stability of the material. Due to the “zero-stress” property, LTO shows minimal degradation over 20,000 cycles, according to the literature [6,7].

Recent studies show that hybrid LTO/carbon anodes can reduce gas formation during battery operation. The incorporation of carbon materials effectively covers the active surface sites on the LTO anode, suppressing gas effects and enhancing battery stability. In addition, carbon acts as a conductive interface, reducing charge transfer resistance and facilitating the diffusion of ions. This improves the anode’s electrical conductivity, diffusion coefficient, and capacity while ensuring its structural stability and safety. Researchers have explored various carbon materials, such as graphene and carbon nanotubes, to achieve optimal performance [8,9,10]. Graphene (744 mAh g^−1^) [11,12,13], carbon nanotubes (CNTs, capacity range: 300~1000 mAh g^−1^) [12], and carbon nanofibers (CNFs—550 mAh g^−1^) [11] generally exhibit higher theoretical capacities than graphite [13]. However, it is important to note that this additional capacity may not be fully reversible and does not exclusively represent the intrinsic capacity of the sp^2^ carbon [14,15]. Instead, it is influenced by the unique structure of each material, which enhances ion transport and prevents the degradation of the material. In general, composites tend to exhibit enhanced properties compared to the original materials, making mixed anodes particularly beneficial, as they leverage the synergistic effects between LTO and carbon [14]. In these composites, the LTO component facilitates faster lithium-ion transport, which then distributes to the carbon sites, enhancing access to ion adsorption sites and thus increasing the overall capacity. Furthermore, the structural properties of carbon play a significant role in improving the performance of LTO-based electrodes. For example, carbon coatings on LTO enhance electrical conductivity and increase the active area for lithium-ion interaction, which is crucial for maintaining capacity during fast charge/discharge cycles [14,15]. Furthermore, studies show that LTO/carbon composites retain a higher percentage of their original capacity over long-term cycling compared to pure LTO electrodes [14,15].

Graphene offers a specific capacity of 744 mAh g^−1^, which is double that of graphite (372 mAh g^−1^) [13]. Graphene oxide has a capacity of approximately 660 mAh g^−1^,though this can vary depending on the composites it forms. In contrast, carbon nanotubes (CNTs) have a theoretical capacity of 1116 mAh g^−1^, four times higher than graphite [12,13,14,15]. CNTs, carbon nanofibers (CNFs), and carbon nanosheets are especially beneficial as anode additives for LIBs due to their excellent electrical conductivity, which facilitates efficient charge transfer [16,17,18,19,20]. CNTs are known for their ability to maintain structural stability over many charge/discharge cycles. On the other hand, CNFs are known for their excellent electrical conductivity and flexibility. When incorporated into LTO, they create a highly conductive three-dimensional network that facilitates electron transfer. This network significantly improves the overall conductivity of the composite, enhancing electrochemical performance during charge and discharge cycles [21]. Additionally, carbon nanosheets provide a large surface area for lithium-ion storage, which is beneficial for improving the capacity of LTO-based electrodes. Their two-dimensional structure allows for more active sites for lithium-ion insertion and during battery operation [19,20].

Furthermore, understanding the interaction between carbon and LTO is essential for optimizing carbon doping or templating processes, as the method of carbon incorporation significantly influences the properties of the final materials. Given the focus of this review on energy storage applications, it is important to clarify that incorporating carbon into LTO during the synthesis process is preferable to the physical mixing of the two precursors [20]. This approach promotes chemical interactions during synthesis, resulting in a more uniform composite and providing better control over key properties such as porosity, active surface area, and functional groups [20]. These characteristics enhance charge transfer and improve overall anode performance.

Another strategy to address the issue of gas generation is the careful selection and investigation of suitable electrolytes. LTO’s compatibility with various electrolyte types offers a significant advantage. Research by Mickey et al. indicates that while a decrease in H_2_ production is associated with an increase in CO_2_, this can lead to higher overall gas production during continuous charging cycles [4]. However, Sato et al. have shown that aqueous electrolytes, such as LiCl and Li_2_SO_4_, when paired with a lithium-ion conductive solid electrolyte (SE) separator and a Zn-coated anode, can effectively suppress H_2_ gas evolution [5]. Recent studies suggest that aqueous electrolytes offer a promising approach for mitigating gas production, providing enhanced safety, lower construction costs, and a reduced environmental footprint.

This review examines the characteristics of LTO and explores strategies for various hybrid LTO/carbon synthesis techniques to improve cycle stability, coulombic efficiency, and safety. It also suggests ways to further improve LTO’s characteristics, focusing on developing sustainable anodes with reduced environmental impact.

## 2. Crystal Structure of LTO

**Figure 1:**
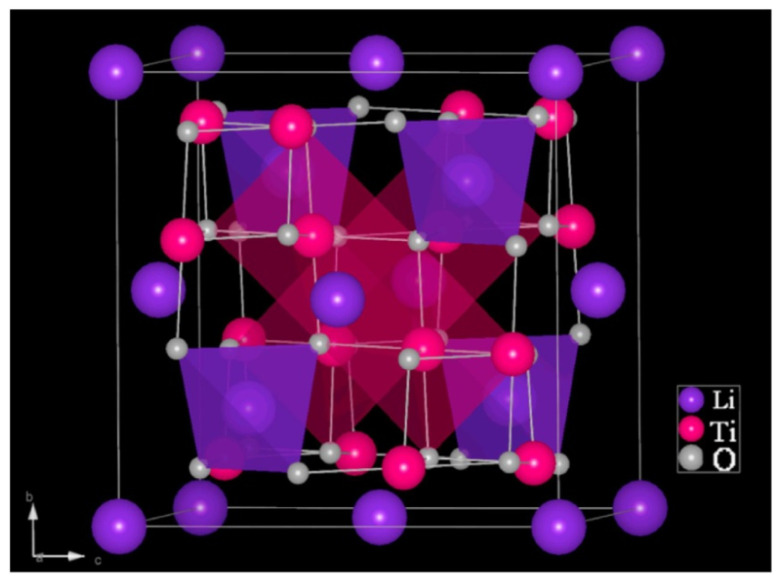
Crystal structure of LTO [22].

Li_4_Ti_5_O_12_ or LTO has emerged as a promising anode material for LIBs due to its unique crystal structure and exceptional electrochemical properties. Its cubic spinel structure (Figure 1) provides a three-dimensional pathway for lithium-ion diffusion, facilitating fast lithium-ion transport kinetics [22].

This unique structure allows lithium ions to be intercalated into and de-intercalated from the LTO lattice with minimal structural changes, resulting in exceptional cycle stability. The electrochemical reaction of LTO as an anode material can be described by Equation (1) [23]:

Li_4_Ti_5_O_12_ + 3Li^+^+ 3e^−^ ⇆ 3Li_2_TiO_3_ + 2Li_2_Ti_3_O_8_ (1)

**Figure 2:**
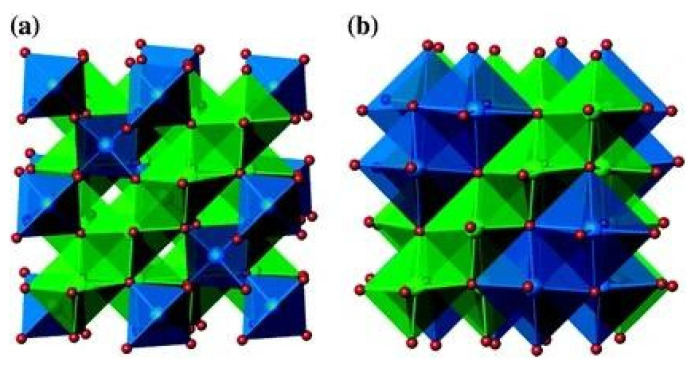
(**a**) Li_4_Ti_5_O_12_ spinel structure and Li_7_Ti_5_O_12_ rocksalt (**b**). Blue octahedra represent Li, and green octahedra represent disordered Li and Ti [31].

This was further validated by Ohzuku et al., who reported that the “zero-strain” insertion/extraction process allows LTO to undergo these processes without significant volume changes (around 0.2%), contributing to its excellent cycle stability [24]. Specifically, LTO’s spinel form features a cubic crystal structure belonging to the Fd3m space group, as per Wyckoff positions in crystallography [24,25]. Oxygen atoms occupy positions 32e, Li atoms are situated in tetrahedral positions 8a, Ti atoms are inpositions 16c, and additional Li atoms fill the remaining vacant 16c sites [25,26], resulting in a phase transformation from the spinel structure of Li_4_Ti_5_O_12_ to the rock-salt structure of Li_7_Ti_5_O_12_ [27,28,29,30]. As illustrated in Figure 2, this structural transformation results in only a slight lattice contraction, from 8.3595 Å in Li_4_Ti_5_O_12_ to 8.3538 Å in Li_7_Ti_5_O_12_, corresponding to a volume reduction of about 0.2%. Due to this negligible volume change, Li_4_Ti_5_O_12_ is widely recognized as a “zero-strain” material, making it highly stable for lithium-ion insertion and extraction. This provides LTO with superior structural stability and guarantees the mechanical integrity of the electrode [31].

## 3. Synthesis Methods

In this section, we will provide a concise overview of the three principal methods for LTO synthesis, as reported in the literature: solid-state reaction, hydrothermal synthesis, and sol–gel synthesis. The aim of this investigation is to identify the most energy-efficient technique that produces a material with optimal properties for superior performance.

According to the literature, the LTO spinel structure has short paths for the transport of lithium ions and electrons that contribute to the fast charging of LIBs. However, the low ionic conductivity of LTO (1.8 × 10^−8^ Sm^−1^ [32]) limits its performance rate. One effective way to address this limitation is to combine LTO with conductive materials (such as carbon nanotubes and reduced graphene oxide) in the synthesis process [8,9,10,32,33,34] to enhance ion and electron flow.

### 3.1. Solid-State Synthesis

**Figure 3:**
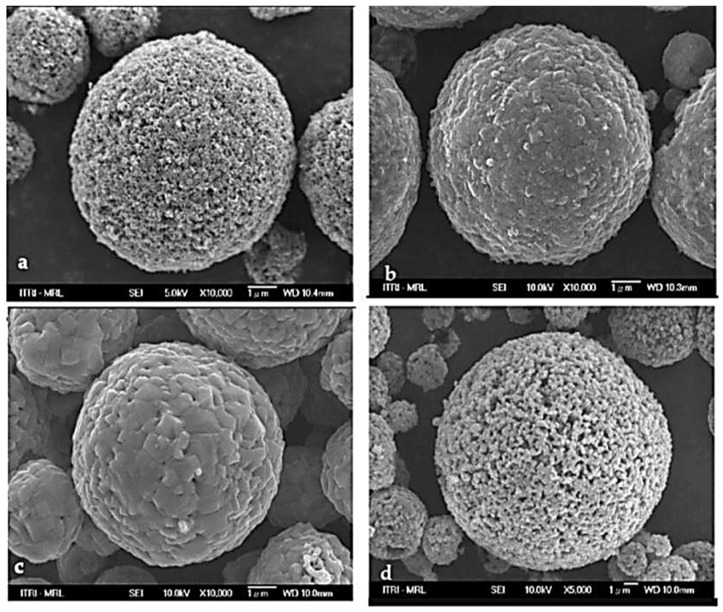
FESEM images of the spray-dried precursor particles comprising (**a**) nano-anatase, (**b**) submicronrutile TiO_2_ particles, (**c**) pyrolyzed D-Li_4_Ti_5_O_12_, and (**d**) P-Li_4_Ti_5_O_12_ [35].

In 1998, D. Peramunage and K. Abraham successfully developed LTO using a two-stage solid-state synthesis method with LiOH and TiO_2_ anatase as precursors [34]. The process began with heating a well-ground stoichiometric mixture of TiO_2_ anatase and LiOH at 800 °C. The resulting product was then ground and sintered to achieve the desired particle size, creating a submicron dispersion of TiO_2_ and Li_2_CO_3_ in hexane. After removing the hexane, the remaining material was calcined at 800 °C, yielding a final mixture that was combined to obtain the stoichiometric Li_4_Ti_5_0_12_. A few years later, in 2008, Hsiao et al. [35] demonstrated that LTO grain size significantly impacts its electrochemical performance. They found that a porous structure with a smaller particle size offers a larger active surface area, enhancing anode performance. In their study, both dense and porous Li_4_Ti_5_O_12_ powders were prepared through a spraying process. This involved dissolving LiOH, TiO_2_, and a 5% weight dispersant (BYK-190 relative to TiO_2_) in distilled ionic water. Two types of TiO_2_ particles were employed, anatase with an average size of 21 nm and rutile at 210 nm, while maintaining a lithium-to-titanium oxide molar ratio of 4:5. The precursor slurries were mixed with alumina balls using a three-dimensional rotary mixer at 300 rpm for 8 h to achieve homogeneity. Once mixed, the slurries were sprayed at 250 °C using a two-liquid nozzle with a spray pressure of 3 kg cm^−2^. The spray-dried powders were then further processed by air-firing at 850 °C for 8 h to give Li_4_Ti_5_O_12_ particles in the spinel phase [35]. The pyrolysis temperature plays an important role in determining the final structure of the materials as it affects the kinetics of the chemical reactions occurring during pyrolysis. A higher temperature generally increased the reaction rates, resulting in more complete reactions and leading to a more homogeneous structure in terms of the particle’s size and structural integrity. Figure 3a,b present FESEM images of two types of powders spray-dried at 250 °C in air. The powders prepared from TiO_2_ anatase nanoparticles show a porous structure (P-Li_4_Ti_5_O_12_), as indicated in Figure 3a. In contrast, the powders derived from submicron TiO_2_ rutile particles display a dense and smooth surface (D-Li_4_Ti_5_O_12_), as shown in Figure 3b [35]. These structures appear to change after pyrolysis at 850 °C for 8 h, as shown in Figure 3c,d. The P-Li_4_Ti_5_O_12_ particles become denser, while the D-Li_4_Ti_5_O_12_ particles, initially dense, show a slight increase in primary size from 210 nm to 220 nm and transform into a more porous structure (Figure 3d) [35]. This research indicates that porous Li_4_Ti_5_O_12_ powders (P-Li_4_Ti_5_O_12,_ derived from TiO_2_ nanoparticles annealed at 850 °C for 8 h) demonstrate superior characteristics compared to dense Li_4_Ti_5_O_12_ powders (D-Li_4_Ti_5_O_12,_ derived from TiO_2_ submicron particles annealed at 850 °C for 8 h). The P-Li_4_Ti_5_O_12_ powders are expected to achieve a higher discharge capacity, owing to their shorter lithium-ion transport paths and higher electronic conductivity. These advantages result from their smaller primary particle size and higher surface area compared to D-Li_4_Ti_5_O_12_.

**Figure 4:**
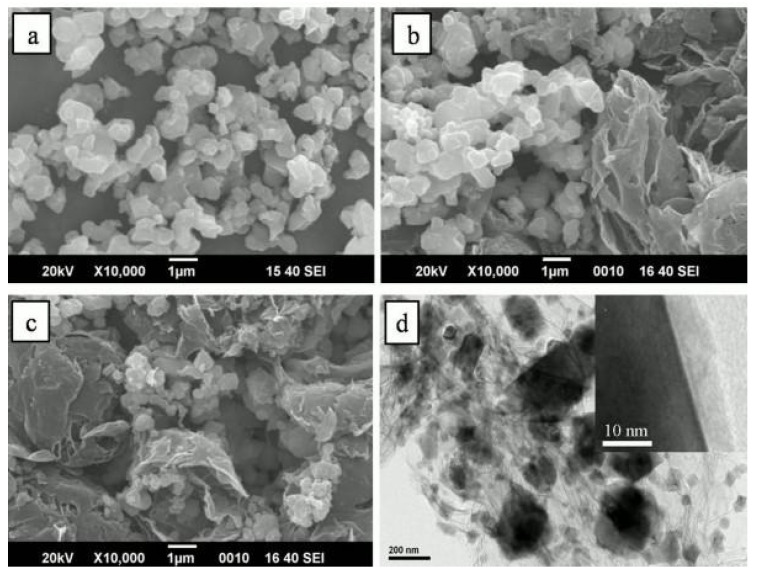
SEM images of (**a**) LTO, (**b**) LTO/graphene, and (**c**) C-LTO/graphene and TEM image (**d**) of C-LTO/graphene [36].

A few years later, in 2013, Guo et al. prepared a homogeneous Li_4_Ti_5_O_12_/graphene composite via an insitu solid-state reaction following carbon pre-coating [36]. In their work, they compared this microstructure with materials without carbon coating. The results showed that carbon coating not only effectively limits the aggregation of Li_4_Ti_5_O_12_ particles but also enhances the combination between Li_4_Ti_5_O_12_ andthe graphene sheets. The precursors used were TiO_2_ anatase and glucose in a 4:1 weight ratio, which were mixed in an ethanol/water solution (10:1 by volume) and stirred for 2 h. Then, the mixture was dried for 10 h in an air-circulating oven at 100 °C. Subsequently, it was heated at 600 °C for 5 h under a N_2_ atmosphere to obtain carbon-coated TiO_2_, with the carbon content in TiO_2_ at approximately 6% *w*/*w*. Graphene, prepared using the Hummer method, was then mixed with Li_2_CO_3_ and the carbon-coated TiO_2_ in hexamethylene. The mixture was subjected to ball milling using a planetary ball mill (Nanjing) at a rotation speed of 200 rpm for 4 h under an inert atmosphere. After drying, it was further heated at 800 °C for 12 h in the N_2_ atmosphere to obtain the carbon-coated Li_4_Ti_5_O_12_/graphene composites (denoted as C-LTO/graphene). In this composite material, the total carbon content, including both the coated carbon and the graphene sheets, was approximately 10%. For comparison, they examined pure Li_4_Ti_5_O_12_ and Li_4_Ti_5_O_12_/graphene uncoated carbon composite (LTO/graphene) [36]. As shown in Figure 4a, the primary LTO particles are 200–800 nm in size and are able to aggregate together. The LTO/graphene image, shown in Figure 4b, indicates that the LTO particles are almost separated by graphene sheets. However, the particle size and degree of aggregation in the LTO/graphene composite remain comparable to those of pure LTO. This indicates that bare LTO struggles to anchor firmly onto the graphene sheets, resulting in aggregation and ultimately an inhomogeneous LTO/graphene composite. In contrast, the images of the C-LTO/graphene composite (Figure 4c,d) demonstrate that the LTO particles are uniformly dispersed within a three-dimensional network created by the graphene sheets, providing a conductive connection between the LTO particles due to the presence of carbon coating [36]. Therefore, carbon coating emerges as essential in preventing the aggregation and coagulation of Li_4_Ti_5_O_12_ particles while promoting their effective integration with graphene sheets, resulting in homogeneous composites. However, no specific details on the synthesis parameters such as the annealing temperature are provided because the focus is mainly on the effect of the carbon coating on the homogeneity of the composite. This approach is anticipated to yield a composite material with excellent rate capability.

### 3.2. Hydrothermal Synthesis

**Figure 5:**
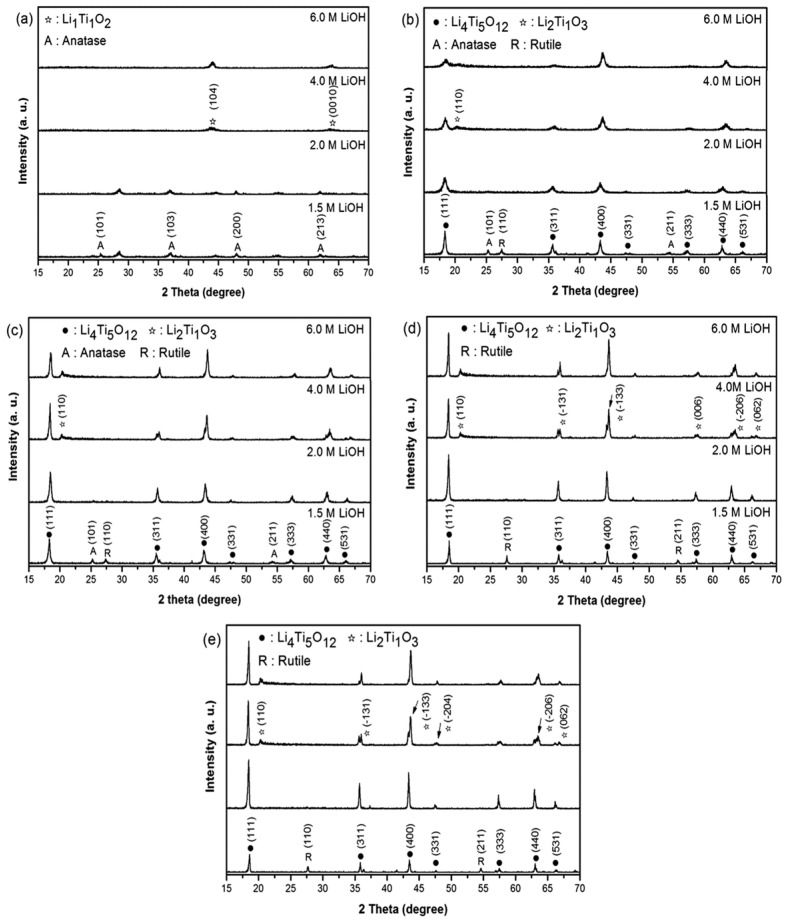
XRD of LTO obtained from reactants with different concentrations of LiOH: (**a**) pristine and those that were subject to heat treatment in air at (**b**) 450 °C, (**c**) 550 °C, (**d**) 650 °C, and (**e**) 750 °C [38].

Another method for synthesizing LTO is the hydrothermal technique. Zhang et al. employed this method using LiOH·H_2_O and titanium tetrabutyl ester (Ti(OC_4_H_9_)_4_) in a Li:Ti molar ratio of 4:5 [37]. The process began by mixing titanium tetrabutyl ester with ethyl alcohol to create Solution A. A 2 M aqueous solution of LiOH was then added dropwise to Solution A while vigorously stirring for 30 min, resulting in a white Suspension B. This mixture was then transferred to a stainless-steel autoclave and heated at 180 °C for 24 h. The resulting precursor was calcined at 500 °C for 10 h to obtain the final Li_4_Ti_5_O_12_ powders. Initially, the precursor exhibited a non-uniform texture, but the Li_4_Ti_5_O_12_ displayed a spherical morphology with diameters ranging from 1 to 5 μm. The microspherical shape enhances the material’s specific surface area, providing additional channels for lithium-ion transport and storage, thereby enhancing electrode performance. A similar conclusion was reached by Wu et al., who demonstrated that the shape and size of the LTO are closely related to the annealing temperature [38]. Their study explored the effects of varying LiOH precursor concentrations and annealing temperatures affecting the morphology and electrochemical properties of LTO synthesized by the hydrothermal method. In their process, LTO with a nanosheet structure was obtained by hydrothermally mixing Ti(OC_4_H_9_)_4_ in ethanol with LiOH, followed by heating at 180 °C for 12 h in a Teflon-lined stainless-steel autoclave. The final products were then heat-treated at 450 °C, 550 °C, 650 °C, and 750 °C for 6 h. XRD analysis, as shown in Figure 5, revealed that an optimal concentration of 2 M LiOH yielded the most favorable results, with clearly defined diffraction peaks indicating well-crystallized samples. The purity of the powders was strongly influenced by both the concentration of the reactants and the annealing temperature. At a 2 M LiOH concentration, the powders primarily consisted of spinel LTO. However, at higher concentrations (4 M and 6 M), additional small peaks corresponding to rutile TiO_2_, anatase TiO_2_, and Li_2_TiO_3_ were observed, indicating incomplete reactions. These results highlight that optimal reactant concentrations promote efficient interactions, leading to complete reactions and the formation of the desired product, whereas concentrations that are too high or too low can result in incomplete reactions or promote the formation of unwanted by-products.

**Figure 6:**
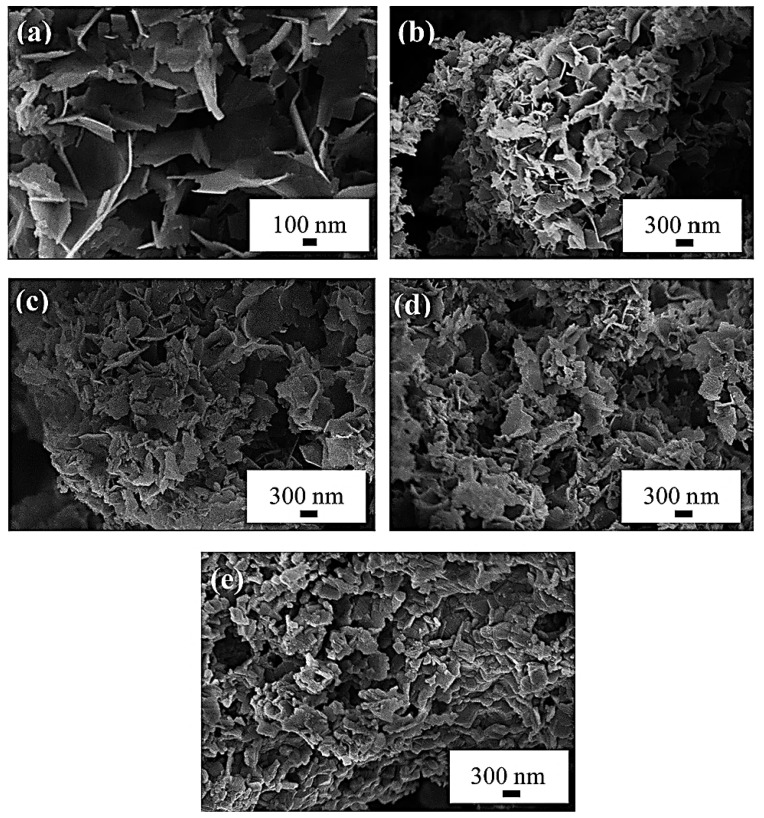
SEM images of (**a**) pristine, (**b**) 450 °C, (**c**) 550 °C, (**d**) 650 °C, and (**e**) 750 °C samples using 2 M LiOH [38].

The study revealed that a single-phase spinel LTO, free of impurities, was successfully prepared in 2 M LiOH and annealed at 450 °C, 550 °C, 650 °C, and 750 °C, resulting in nanosheets, as shown in the SEM images in Figure 6. The SEM images in Figure 6b–e illustrate that the nanosheet structure is preserved after annealing. Although the nanosheets retain their sheet-like morphology as the annealing temperature increases, they also become thicker compared to the initial powder. However, the pyrolysis that occurs during the annealing process induces some structural damage to the LTO nanosheets. These results underscore the critical role that the annealing temperature plays in determining the thickness and shape of the nanosheets [38]. It is important to recognize that there is a threshold for annealing temperature, as excessively high temperatures can lead to damage, likely due to particle aggregation. At high temperatures, the increased thermal energy allows atoms and particles to move more freely, facilitating the migration and subsequent aggregation of particles into larger agglomerates. Additionally, high temperatures facilitate the coalescence of individual particles, leading to the fusion and aggregation of nanosheets. Accordingly, this research highlights the optimal conditions for producing lithium titanate (LTO) nanosheets with enhanced morphology and structure. Using a 2 M LiOH aqueous solution and heat-treating the nanosheets at 550 °C, the study achieves highly crystalline spinel LTO nanosheets with a high surface area and short lithium-ion diffusion paths. The fine morphology of the nanosheets, which remain intact after heat treatment, can enhance their electrochemical properties by providing additional transport channels for the insertion of lithium ions.

**Figure 7:**
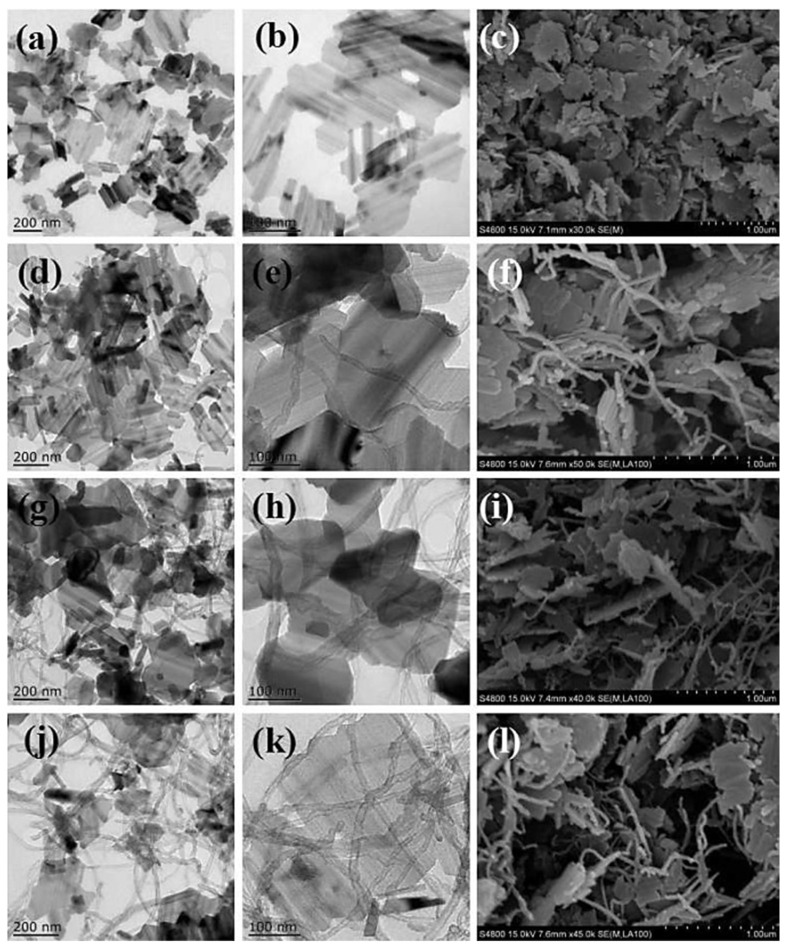
TEM and SEM images of (**a**–**c**) LTO NSs and LTO NS/CNT composites with (**d**–**f**) 5%, (**g**–**i**) 7.5%, and (**j**–**l**) 10% CNTs [8].

There are also numerous studies regarding the integration of LTO with carbon materials for the enhancement of their performance. For instance, Zhang et al. prepared Li_4_Ti_5_O_12_ nanosheets (LTO NSs)/carbon nanotubes (CNTs) using the hydrothermal method, finding that the addition of CNTs improved the rate performance of LTO NSs [8]. In their process, 1.7 g tetrabutyl titanate (TBT) and 0.204 g LiOH·H_2_O, along with varying amounts of CNTs, were mixed in 20 mL of ethanol at room temperature and stirred for about 12 h. Subsequently, 25 mL of deionized water was added, and the solution was stirred continuously. The mixture was then transferred toa Teflon-lined stainless-steel autoclave and heated at 180 °C for 36 h. Afterwards, the white powder formed at the bottom of the reactor was collected by centrifugation, washed three times with ethanol, and dried in an oven at 60 °C for 8 h. To generate LTO NS/CNT composites, the precursor was heated at 700 °C for 6 h in a horizontal tubular furnace under an Ar atmosphere. For comparison, LTO NSs without CNTs were synthesized using the same procedure [8]. The study highlights that pure LTO exhibits two-dimensional nanolayers with an irregular shape, as shown in Figure 7a,b [8]. In contrast, the LTO NSs, which have an average diameter of 300 nm, exhibit well-crystallized structures. The SEM image in Figure 7c shows that after calcination, the LTO NSs have a thickness of 20 nm and an average size of 300 nm. The TEM images in Figure 7d–f reveal that the LTO NS/5%-CNT composites maintain their sheet-like morphology, despite the presence of CNTs. As the CNT content increases (from 7.5% to 10%), the CNTs become more widely dispersed across the LTO NSs’ surfaces (Figure 7h,k), forming conductive nanowires that interconnect the LTO NSs and establish a network structure (Figure 7g,j). This uniform CNT distribution enhances the contact area with lithium ions, facilitating rapid electron transfer. SEM images further corroborate the morphology of the LTO NS/CNT composites (Figure 7f,i,l), where CNTs are embedded within the LTO NS layers [8]. SEM images further confirm the morphology of the LTO NS/CNT composites. In Figure 7f,i,l, CNTs are incorporated into the layers of LTO NSs, enhancing the composites’ electronic transmission capabilities and improving lithium-ion diffusion between the LTO NSs.

**Figure 8:**
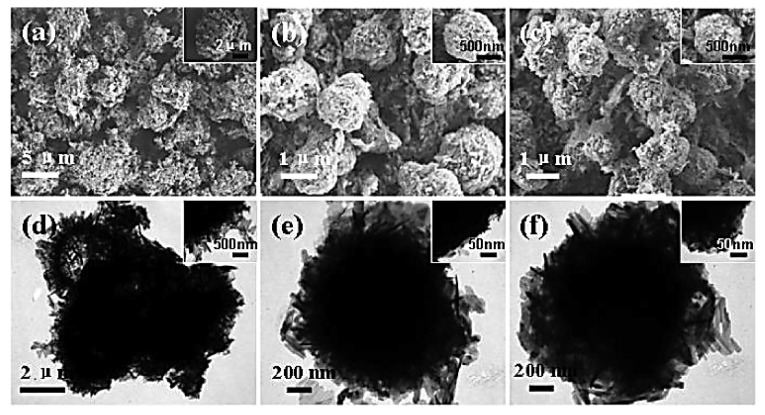
SEM and TEM images of pristine LTO (**a**,**d**), 1.0 wt% rGO-coated LTO (**b**,**e**), and 3.0 wt% rGO-coated LTO (**c**,**f**) [10].

On the other hand, Zhang et al. developed a composite material consisting of Li_4_Ti_5_O_12_ microspheres and reduced graphene oxide (rGO) [10]. Their approach involved the hydrolysis of titanium butoxide, followed by a 36h hydrothermal reaction at 180 °C using a TiO_2_ precursor, GO, and LiOH. The resulting composite was then heat-treated at 600 °C in an Ar atmosphere. Specifically, TiO₂ powder was synthesized by hydrolyzing tetrabutylortho-titanate (TBOT). A solution of 2 mL TBOT in 8 mL ethanol was slowly added to a solution of PVP (0.02% *w*/*w*) in 100 mL ethanol with 1 mL water. After stirring, the mixture underwent hydrothermal treatment at 80 °C for 2 h. The resulting TiO_2_ precipitate obtained was washed, air-dried, and mixed with LiOH solution, maintaining a Ti-to-Li molar ratio of 5:4.5. This mixture was subjected to a 36h hydrothermal treatment at 180 °C, followed by calcination at 600 °C under an Ar atmosphere to obtain spinel LTO. GO was prepared by the modified Hummers method and added to the TiO_2_-LiOH mixture prior to hydrothermal treatment, with 1.0 wt% and 3.0 wt% being incorporated into the GO/LTO composites. Figure 8 shows the SEM and TEM images of the microstructures of the original LTO and LTO particles coated with 1.0 wt% and 3.0 wt% rGO [10]. The original LTO particles exhibit an irregular nanoplatelet shape with a non-uniform size distribution (Figure 8a). In contrast, the LTO particles with 1.0 wt% rGO and 3.0 wt% rGO coatings exhibit a spherical morphology (Figure 8b,c). The TEM images in Figure 8e,ffurther reveal that the LTO particles with both rGO coatings maintain a similar microstructure, with an almost spherical shape and an approximate diameter of 1.6 μm. Moreover, the surface of the LTO particles with 3.0 wt% rGO appears relatively compact and uniform [10], which could potentially enhance the kinetics of lithium ions.

### 3.3. Sol–Gel Synthesis

**Figure 9:**
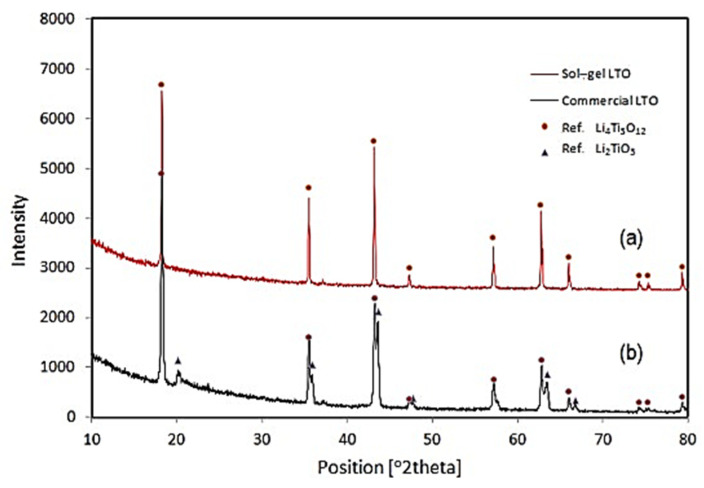
XRD pattern from (**a**) sol–gel and (**b**) commercial LTO [40].

**Figure 10:**
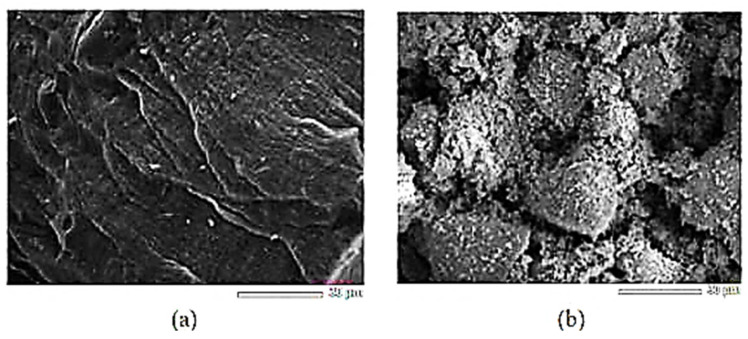
SEM images of (**a**) sol–gel and (**b**) commercial LTO. The bar is equal to 30 μm [40].

Sol–gel is another method for synthesizing LTO, known for generating smaller and more homogeneous particles [21]. In this process, chelating agents (such as oxalic acid) are crucial, as they significantly influence the particle size [39]. By binding to metal ions, these agents influence nucleation, which directly affects the formation of nuclei and the distribution. They also regulate the growth rate of these particles by stabilizing or destabilizing ion species, ensuring a more uniform particle size and distribution. Purwamargapratala et al. employed the sol–gel method to synthesize LTO, utilizing titanium butoxide as the titanium source and lithium dihydrate acetate as the lithium source [40]. Lithium dihydrate acetate was dissolved in a mixture of ethanol and deionized water at a ratio of 5:1. Oxalic acid was then added in a 1:1 ratio with ethanol, and the solution was stirred for 4 h before being allowed to stand for 12 h. The mixture was dried at 80 °C for 6 h, crushed, and subjected to a heat treatment sequence: 200 °C for 30 min, 500 °C for 30 min, and finally calcined at 800 °C for 30 min to 1 h. The sol–gel-synthesized LTO was compared with commercially available Li_4_Ti_5_O_12_. XRD analysis was performed using the quantitative phase analysis (QPA wt%) method, utilizing Highscore Plus software [40], in which the weight percentage (wt%) of different crystalline phases present in the mixture was determined. This analysis revealed that the sol–gel LTO achieved 99% purity, consisting primarily of Li_4_Ti_5_O_12_with only 1% Li_2_TiO_3_. In contrast, the commercial LTO consisted of 57.7% Li_4_Ti_5_O_12_ and 42.3% Li_2_TiO_3_ (Figure 9) (Table 1) [39]. The sol–gel-synthesized LTO (Figure 10a) shows a sheet-like morphology with an average size of 1 μm, a structure that enhances ion kinetics by promoting the diffusion of lithium ions. In contrast, the commercial LTO particles (Figure 10b) show a grainy texture with much larger particles, averaging 30 μm in size [40]. This comparison shows that sol–gel synthesis not only yields higher-purity LTO but also produces a sheet-like particle structure, which, as highlighted in the introduction, can significantly enhance overall conductivity.

**Figure 11:**
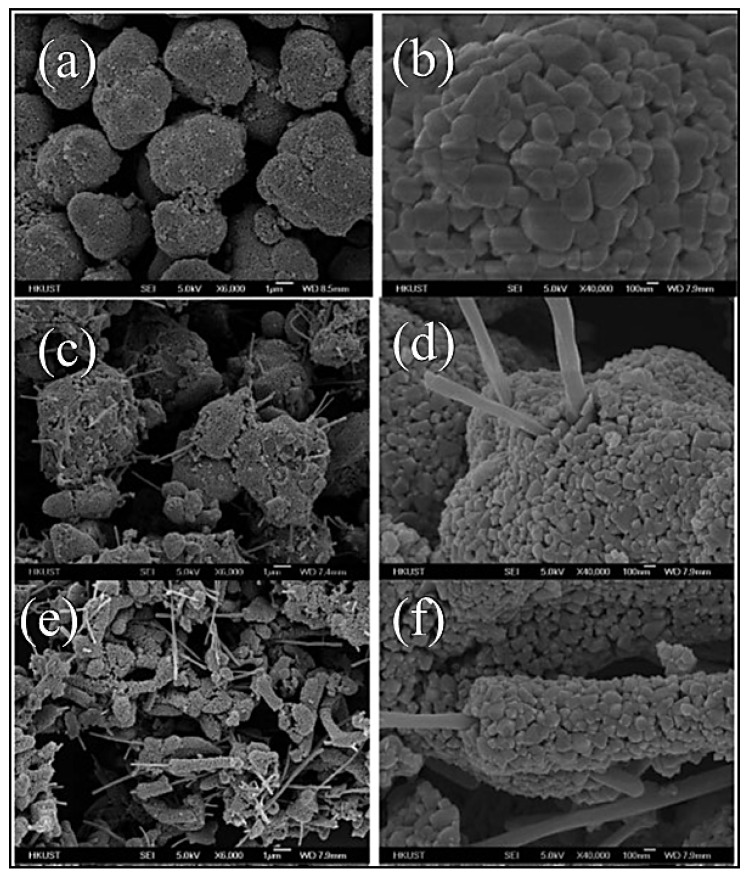
SEM images of (**a**,**b**) LTO, (**c**,**d**) LTO–CNF-1 (5.9% CNFs), and (**e**,**f**) LTO–CNF-2 (11.1% CNFs) composites. The bar is equal to 1 μm for the Figure 3a,c,e and 100 nm for the Figure 3b,d,f. [9].

In a study conducted in 2012, Zhang et al. developed a composite powder of LTO and carbon nanofibers (CNFs) using the sol–gel method [9]. The base material was pure LTO powder, specifically Li_3.9_Sn_0.1_Ti_5_O_12_ with a minor Sn^2^⁺ impurity, intended for use in LIBs. The CNFs, ranging from 10 to 20 mm in length and averaging 150 nm in diameter, were dispersed in ethanol using ultrasound. Titanium isopropoxide was then added to the solution, followed by dehydrated lithium acetate and tin (II) chloride, all mixed under vigorous stirring. To account for lithium evaporation during high-temperature synthesis, an excess of 10 mol % of lithium was added. The resulting gel was dried at 60 °C for 24 h and calcined at 800°C for 12 h in a nitrogen atmosphere to produce LTO-CNF composite powders. Two variants were prepared: LTO-CNF-1 (5.9% CNFs) and LTO-CNF-2 (11.1% CNFs). For comparison, a batch of LTO powder was synthesized without CNFs [9]. Figure 8 displays SEM images that highlight the distinct characteristics of the composite powders based on the CNF content. Initially, the pure LTO particles exhibit spherical secondary structures formed from primary particles (ranging from 100 to 500 nm), with small pores visible (Figure 11a,b). Upon adding CNFs, the morphology of the secondary particles changes. At 5.0% *w*/*w* CNFs, the secondary particles acquire an irregular, sea urchin-like appearance, with multiple CNF rods penetrating them (Figure 11c,d). With a 10% *w*/*w* CNF content, the particles elongate into a corn-shaped structure, with CNFs embedded longitudinally (Figure 11e,f). These morphological changes are attributed to the self-assembly of primary LTO particles around the CNFs during calcination, which limits primary particle growth and inhibits further growth [9]. This leads to the conclusion that by adding conductive materials such as CNFs, a larger active surface area is achieved, expected to enhance the electrochemical performance due to the better ion mobility in the material structure.

### 3.4. Overview

In summary, the synthesis method has a profound impact in determining the structural and morphological characteristics of LTO. While solid-state reaction methods are cost-effective, they require high calcination temperatures (700–800 °C) and prolonged reaction times, which can lead to particle aggregation, negatively impacting adhesion. On the other hand, the hydrothermal method yields smaller, more reactive particles, offering advantages over the solid-state method. Additionally, the sol–gel method, using chelating agents such as oxalic acid, achieves high purity (99% Li_4_Ti_5_O_12_) and smaller particles, offering enhanced control over the morphology and particle size—factors crucial to the material’s performance.

Overall, hydrothermal synthesis is particular effective, enabling the production of LTO nanosheets at low temperatures (i.e., 180 °C) compared to the other methods, thereby reducing the environmental impact. A comparison of porous versus dense Li_4_Ti_5_O_12_ powders reveals that porous structures, derived from TiO_2_ nanoparticles as precursors, can offer improved electrochemical performance due to enhanced lithium-ion diffusion. Incorporating graphene oxide further enhances the morphology of LTO composites (i.e., smaller size thus larger active surface area), expected to result in increased specific capacitance and cyclic stability, as will be discussed below.

As summarized in Table 2, spherical structures combined with conductive carbon networks offer a larger active surface area, expected to boost performance.GO has a high surface area and a two-dimensional layered structure, creating a broader and more effective contact area with LTO particles [41,42,43]. This structure enhances electron transfer and provides abundant sites for lithium-ion interaction. In contrast, the one-dimensional forms of CNTs and CNFs offer comparatively less surface area for direct contact with LTO particles [42]. Additionally, GO’s oxygen functional groups improve its dispersion in aqueous or other polar solutions, leading to more homogeneous composite formation with LTO. This characteristic also enhances GO’s compatibility with environmentally friendly processes [43].

## 4. Performance

A battery’s performance is directly related to the structure, morphology, and properties of its components. These characteristics, in turn, are influenced by how the components are assembled, the process parameters (such as temperature and precursors), and any modifications they undergo, including the incorporation of various types or percentages of carbon materials.

**Figure 12:**
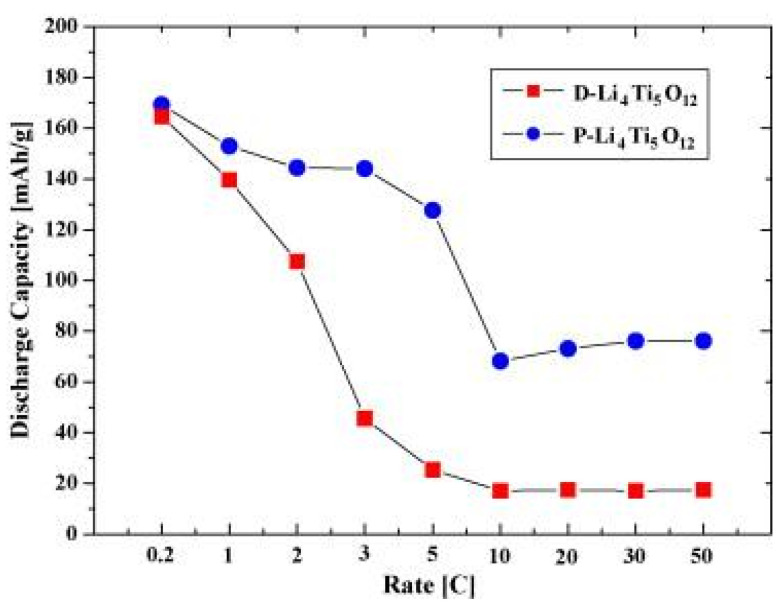
Discharge capacities of the dense LTO (D-Li_4_Ti_5_O_12_) and porous LTO(P-Li_4_Ti_5_O_12_) as a function of discharge rate at a constant charge rate of 0.2C (cut-off voltages: 0.5–2.5 V) [35].

An initial study in 1998 reported the electrochemical behavior of LTOs synthesized through a solid-state reaction [34]. They were tested in lithium-ion cells using 1 M LiPF_6_ electrolyte solution in a 1:1 mixture of EC:PC with carbon as a conductive additive and polyacrylonitrile (PAN)-based polyvinylidene fluoride (PVDF) as a binder. The LTO composite electrode exhibited a capacity of 160 mAh g^−1^ at C/20 to C/30 rates, which was close to the theoretical value (~175 mAhg^−1^) [2]. For these electrochemical measurements, carbon SuperP and KS-6 were used as conductive additives, while PVDF (10% *w*/*w*) was dissolved in N-methyl-2-pyrrolidone (NMP) as a binder. The results showed that LTO particles at the microscale, due to their large active surface area, enhance the diffusion of lithium ions and electrons. A few years later, in 2008, it was shown that the pyrolysis temperature affects the morphology and structure of LTO [35]. As the temperature increased from 500 °C to 800 °C, the structure transitioned from dense to porous, with the porous form exhibiting superior electrochemical behavior due to its larger active surface area. More specifically, the discharge capacities of porous Li_4_Ti_5_O_12_were 144 mAh g^−1^, 128 mAh g^−1^, and 73 mAh g^−1^ at discharge rates of 2C, 5C, and 20C, respectively (cut-off voltages: 0.5–2.5 V), as shown in Figure 12. The corresponding values for dense Li_4_Ti_5_O_12_were 108 mAh g^−1^, 25 mAh g^−1^, and 17 mAh g^−1^. The higher capacities of porous Li_4_Ti_5_O_12_ at high charge/discharge rates were attributed to the shorter Li-ion transport path and higher electronic conductivity, both resulting from the increased active surface area compared to that of dense Li_4_Ti_5_O_12_ [35].

**Figure 13:**
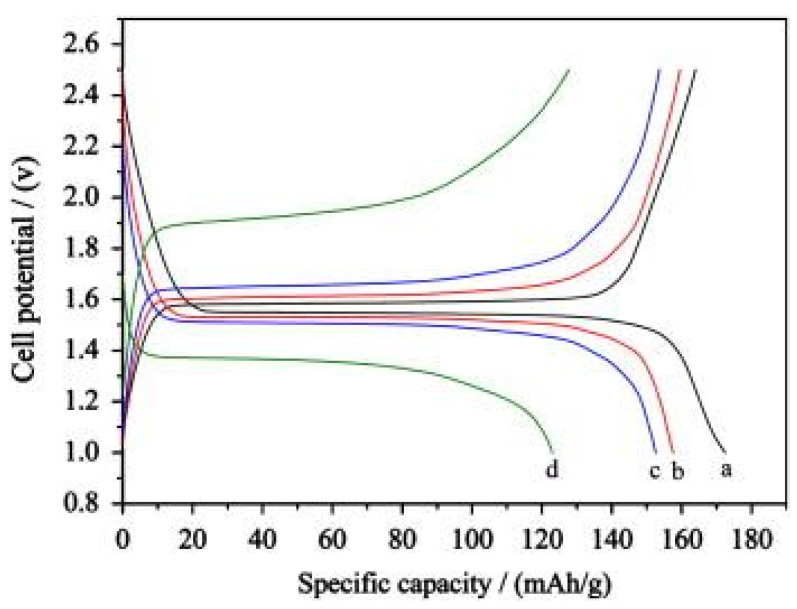
Charge/discharge curves of Li_4_Ti_5_O_12_ for (**a**) 0.1C, (**b**) 0.5C, (**c**) 1.0C, and (**d**) 5C [37].

On the other hand, a study conducted in 2013 [34] reported the synthesis of Li_4_Ti_5_O_12_ nanosheets of a few nanometers in size, through a lower annealing temperature of 500 °C compared to the solid-state reaction synthesis in which higher temperatures (around 800 °C) are commonly used. For the electrochemical measurements, a 1 M LiPF_6_ solution in EC:DMC at a ratio of 1:1 was used as an electrolyte. The charge/discharge tests were carried out within a cut-off voltage of 2.5 to 1.0 V. The results indicated that the synthetic powders have excellent rate capability and cycling performance. The first discharge capacity at 0.1C was 172.5 mAh g^−1^, approaching the theoretical capacity of 175 mAh g^−1^, as shown in Figure 13 [37].

**Figure 14:**
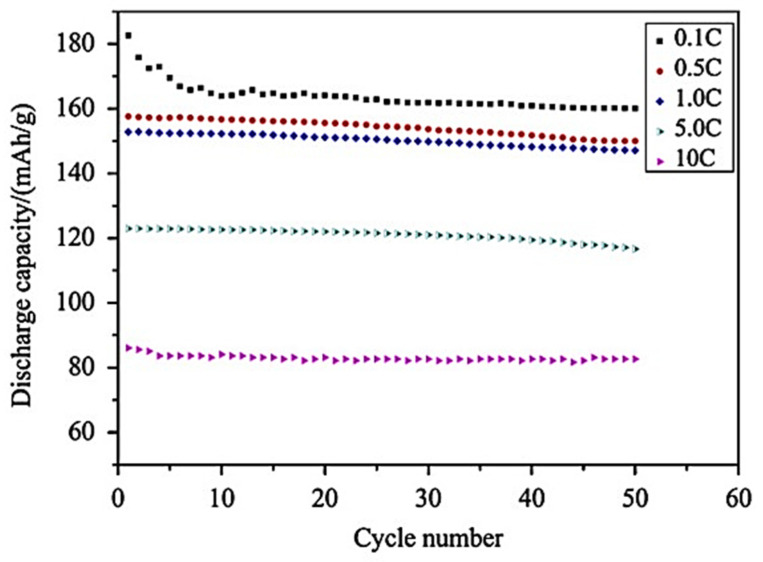
Cycling performances of Li_4_Ti_5_O_12_at different rates [37].

This structure significantly increases the specific surface area, facilitating enhanced transport channels of lithium ions. Consequently, the interface between the electrolyte and the electrode material is improved, reducing irreversible capacity loss and enhancing electrochemical properties such as the specific capacitance and cycling performance. Cycle performance data, shown in Figure 14, highlight the excellent capacitance retention, with over 92% after 50 cycles at 0.1C and maintaining high retention rates at 0.5C and 1C [37].

The above electrochemical measurements indicate that the higher surface area promotes ion diffusion and electron transfer. This conclusion was further supported by a study conducted in 2015 [39], which opted for the hydrothermal synthesis of LTO over the solid-state reaction, known for multiple processing steps and high-temperature requirements. In this study, carbon black was used as a conductive agent, poly-acrylic acid (PAA) served as a water-based binder, and 1 M LiPF_6_ in EC:DEC (1:1) was utilized as an electrolyte [39]. The researchers determined that an annealing temperature of 550 °C for 6 h yielded optimal results (i.e., high surface area), achieving a specific capacity of 175 mAh g^−1^ under different current rates when the cut-off voltage was increased from 0.8 to 2.5 V [38]. From the above study, it is worth noting that with higher temperature treatments (750 °C) damage was caused to the material due to particle aggregation, which verifies that lower temperatures favor crystallinity and promote smaller particle sizes. These characteristics contribute to a larger active surface area, facilitating the insertion and extraction of lithium ions. Similar findings were observed in a recent study, in 2019, in which LTO was synthesized by the sol–gel method at an annealing temperature of 500 °C for 30 min [34] instead of the 6 h duration previously mentioned [38]. The resulting smaller particle size (1 µm) compared to the commercial one (30 µm) was directly linked to improved electrochemical performance. The sol–gel-synthesized LTO showed a capacity of 170 mAh g^−1^ compared to the commercial one, which had 135 mAh g^−1^ in the first charging cycle, verifying that smaller average particle sizes enhanced electrochemical performance. These studies highlight that the capacities reported for the first charging cycles reflect the poor stability of the pure LTO [40]. However, various studies have demonstrated that incorporating conductive carbon materials can significantly enhance the stability of the pure LTO, effectively addressing this challenge.

**Figure 15:**
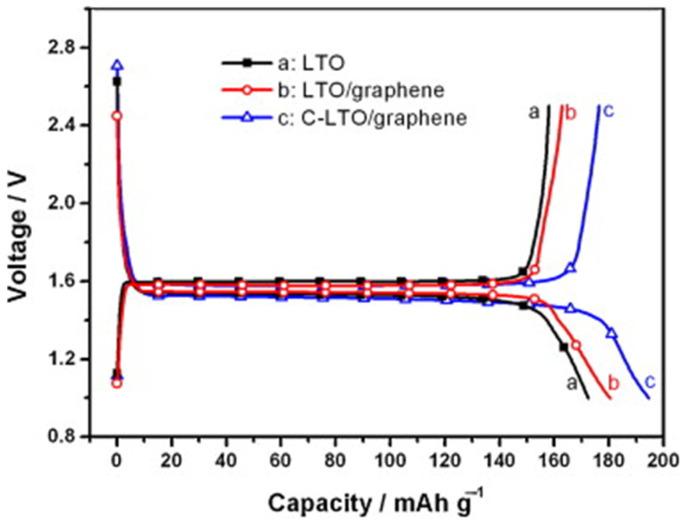
Charge/discharge curves of LTO, LTO/graphene, C-LTO/graphene samples at a constant charge/discharge rate of 0.2C (cut-off voltages: 0.5–2.5 V) [36].

**Figure 16:**
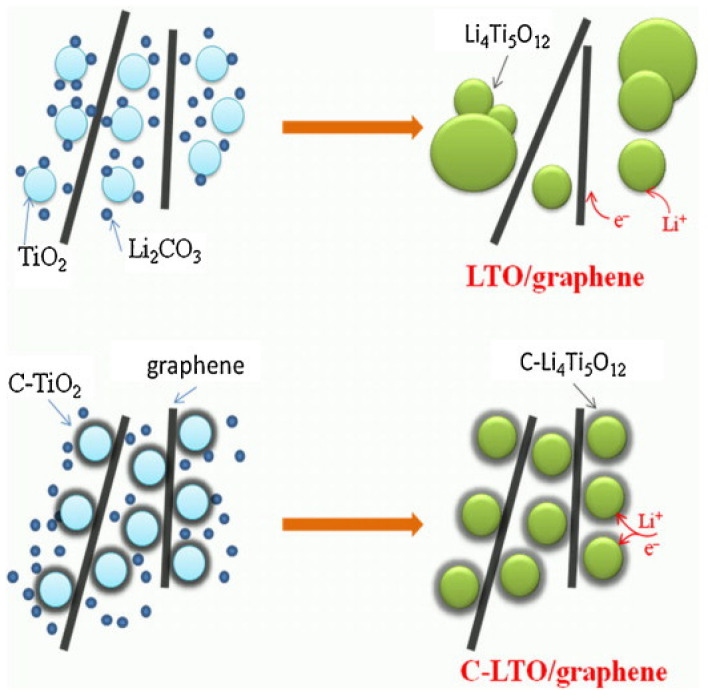
Schematic illustration of the carbon coating effects in LTO/graphene composites [36].

A study conducted in 2013, which compared pure LTO with LTO doped with graphene sheets (LTO/graphene) and carbon-coated LTO doped with graphene sheets (C-LTO/graphene), both synthesized via a solid-state reaction, verified the performance enhancement after carbon coating [36]. For the electrochemical measurements, a 1 M LiPF_6_ electrolyte in EC:DEC (1:1 *w*/*w*) was used, with a current rate of 0.2C and a cut-off voltage of 2.5 V–1.0 V. The charge/discharge tests showed specific capacities of 158 mAh g^−1^ and 163 mAh g^−1^ for pure LTO and the LTO/graphene composite. However, the C-LTO/graphene composite exhibited a higher specific capacity of 177 mAh g^−1^, surpassing the theoretical capacity of LTO (175 mAh g^−1^), as shown in Figure 15 [36]. This increased capacity was attributed to the increased surface area provided by both the carbon coating and graphene sheets (Figure 16), allowing more electrons to be stored within the electrode material, resulting in an increase in capacity. Additionally, the carbon coating prevented the aggregation of LTO particles, maintaining a well-dispersed structure and ensuring the more efficient utilization of the active material [36]. Finally, this uniform dispersion and the close interaction of LTO particles in the graphene sheets increased the electrical conductivity and,as a consequence, the capacity of the composites, as shown in Figure 16.

In 2015, LTO microspheres coated with rGO were synthesized by low-temperature hydrothermal synthesis (180 °C for 36 h) and subsequently electrochemically evaluated in continuous charge/discharge cycles [10]. The results revealed that pure LTO exhibited a capacity of 168 mAh g^−1^ during the first cycle at a rate of 0.5C, which decreased to 135 mAh g^−1^ at 100 cycles. In contrast, the LTO/rGO composite showed much better stability, having an initial capacity of 167 mAh g^−1^ which was maintained at 162 mAh g^−1^ over 100 continuous cycles [10]. Increasing the rate to 15C, the discharge capacity of pure LTO dropped to 48.1 mAh g^−1^ after 150 cycles, while the rGO-coated LTO retained a capacity of 87.5 mAh g^−1^ under the same conditions. This clearly indicated that the presence of rGO enhanced the capacity retention performance of LTO [10]. One year later, in 2016, LTO nanosheets combined with carbon nanotubes were synthesized using facile hydrothermal synthesis, demonstrating excellent lithium storage performance compared to pure LTO NSs as anode materials in LIBs [8]. Electrochemical measurements showed that at a high rate of 5 A g^−1^, the resulting composite LTO nanosheets/CNTs exhibited remarkable rate capability, achieving a reversible capacity of 145 mAh g^−1^ in the first cycle and maintaining 135 mAh g^−1^ at a charge/discharge rate of 2 A g^−1^ even after 1000 cycles, making it a promising anode material for LIBs [8].

**Figure 17:**
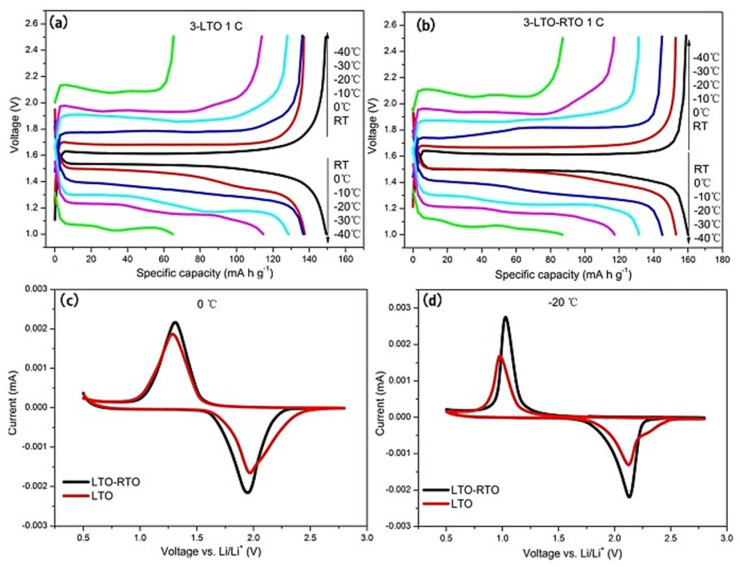
Charge/discharge curves of the LTO (**a**) and LTO–RTO (**b**) electrodes; CV curves of the LTO–RTO and LTO electrodes at 0 °C (**c**) and −20 °C (**d**) [44].

In order to fully evaluate the electrochemical behavior of the LTO anode, the effect of temperature on the electrolyte should be included in the study. Recent research by Huang et al. has shown that the degradation of Li_4_Ti_5_O_12_ anodes at low temperatures is attributed to increased electrolyte viscosity, reduced ionic conductivity, and changes in crystal structure that inhibit the diffusion of lithium ions, leading to reduced electrochemical performance [44]. These factors cause the lithium-ion diffusion pathway to become more energy-intensive. Although this research does not describe in detail the degradation mechanisms at high temperatures, similar issues, such as increased side reactions and structural changes, could affect the performance [44]. Cyclic stability studies focused on low-temperature conditions, particularly between room temperature and −40 °C, demonstrating the superior performance of LTO-RTO (LTO–rutile–LTO composite) electrodes compared to pure LTO electrodes, suggesting their suitability for extreme low-temperature environments. More specifically, to further investigate the dynamics of lithium-ion diffusion in bulk materials, the researchers measured the voltage difference between the charge and discharge (ΔE) and the voltage difference between the oxidation and reduction (ΔV) peaks [44]. Figure 17a,b show the charge/discharge voltage curves for the LTO and LTO-RTO electrodes at 1C from room temperature to −40 °C, while Figure 17c,d show the CV curves for the LTO and LTO-RTO electrodes at 0 °C and −20 °C. As the temperature decreased, both the LTO and LTO-RTO electrodes exhibited an increase in ΔE, indicating higher polarization and reduced lithium-ion diffusion potential. The ΔV value for LTO-RTO was lower than that forLTO, indicating that LTO-RTO electrodes have superior lithium-ion diffusion potential compared to LTO electrodes. The absence of cyclic stability studies at high temperatures suggests a potential area for further research to comprehensively evaluate the performance of the materials over a wider range of operating conditions [44].

In summary, as indicated in Table 3, the electrochemical properties of LTO can be modified depending on the synergy of morphology and the addition of carbon materials. The combination of LTO with conductive carbon materials, such as rGO and CNTs, enhances the capacity retention performance and provides sufficient stability of the anode in continuous charge and discharge cycles, allowing it to maintain its initial capacity even after 1000 cycles. Collectively, these findings position LTO as a promising candidate material for LIB anodes.

## 5. Perspectives

One possible way to strengthen sustainability is the utilization of environmentally friendly carbon sources such as biochar. Unlike traditional methods, such as synthesizing graphene oxide, biochar production has a lower energy footprint and generates no toxic by-products. Biochar stands out as an alternative to traditional carbon materials such as graphite and carbon black, offering a unique structure and properties beneficial to lithium-ion batteries. Its porous structure, shaped by the pyrolysis temperature, along with its high aromatic carbon content, contributes to both stability and electrochemical performance. With a surface area ranging from 100 to 3000 m^2^/g depending on raw material and pyrolysis conditions, biochar competes well with other carbon materials. Graphite, with its well-defined layered structure, and carbon black, consisting of small aggregates of carbon particles, each offer their own advantages in morphology. However, they lack the environmental benefits associated with renewable raw materials [45,46].

Biochar can achieve electrical conductivity levels comparable to commercial carbon black such as Super P, making it a suitable conductive additive for batteries. While the initial capacity of biochar may be lower than optimized graphite, its specific compositions show promising capacities ranging from 150 to 400 mAh g^−1^ during initial cycles [47]. Ongoing research into the properties and synthesis methods of biochar could further enhance its viability as an electrode material for future energy storage applications [47,48].

Another challenge associated with LTO anodes is the emission of gases such as CO_2_ when organic electrolytes are used. While organic electrolytes remain the most widely used with LTO anodes, exploring alternative options is essential to improve battery safety and performance. Aqueous electrolytes, which are non-flammable and environmentally friendly, area promising option, despite facing challenges such as a narrow window of electrochemical stability and insufficient SEI formation [49]. To address these issues, innovative approaches, such as super-concentrated aqueous electrolytes and water-in-salt formulations, are being developed to overcome these challenges [50]. Other alternatives, such as ionic liquids, polymer electrolytes, and gel electrolytes, offer advantages like broader electrochemical stability and increased safety [51]. However, these solutions pose challenges for the research community, particularly due to their limited operational voltage range, which is influenced by titanium salts that can induce water hydrolysis. If successfully developed, these approaches could help resolve energy storage issues, making them increasingly relevant in today’s landscape.

## 6. Conclusions

The synthesis of LTO as an anode material for LIBs has been extensively studied by various methods, including solid-state, hydrothermal, and sol–gel synthesis. The choice of synthesis method and precursors significantly affects the morphology, particle size, and electrochemical performance of the final LTO material. Hydrothermal synthesis is particularly valued for its cost-effectiveness and its ability to control particle morphology, while sol–gel combined with block-copolymer templates can create mesoporous structures that enhance conductivity. Carbon additives play a crucial role in improving LTO’s electronic conductivity, with carbon nanotubes (CNTs) and graphene being particularly effective. CNTcoatingsareconducive to improving the rate and reversibility performance, but graphene oxide coatings significantly improve the electrochemical properties. This is attributed to their high surface area and two-dimensional layered structure, which provide a broader and more effective contact area with LTO particles. Additionally, the presence of oxygen functional groups in graphene oxide makes it a suitable candidate for use in both organic and aqueous media. Despite the challenges of low intrinsic conductivity, these methods and additives collectively enhance LTO’s performance, making it a promising anode material for high-rate charge/discharge applications. In the context of this review paper, the incorporation of conductive carbon materials (e.g., CNTs, rGO) into LTO can boost conductivity through an increase in the active surface area and the facilitation of lithium-ion transport. In addition, the use of aqueous electrolytes is made necessary to ensure the safety of lithium-ion batteries, addressing one of their primary concerns. Overall, optimizing the synthesis method, selecting appropriate carbon additives, and using aqueous media are essential to attain the desired morphology, purity, and electrochemical properties for high-performance sustainable LTO anodes in lithium-ion batteries.

## Figures and Tables

**Table 1:** X-ray refinement results of sol–gel and commercial LTO [40].

Sample	Chemical Formula	Space Group	QPA wt%	Crystallite Size (nm)
Sol–gel LTO	Li_4_Ti_5_O_12_	F d-3m	99.0	561.50
	Li_2_TiO_3_	C 1 2/c 1	1.0	855.03
Commercial LTO	Li_4_Ti_5_O_12_	F d-3m	57.7	396.42
	Li_2_TiO_3_	C 1 2/c 1	42.3	383.30

**Table 2:** Different synthesis methods and parameters affecting LTO’s morphology.

Materials	Synthesis Method	Reaction Temperature	Annealing Temperature	Sample Morphology	Reference
LTO	Solid-state reaction	850 °C		Spherical	[33]
LTO/graphene	Solid-state reaction	850 °C for 12 h		Network structure	[35]
LTO	Hydrothermal	180 °C for 24 h	500 °C for 10 h	Spherical	[36]
LTO	Hydrothermal	180 °C for 12 h	550 °C for 6 h	Nanosheets	[37]
LTO/ CNTs	Hydrothermal	180 °C for 36 h	700 °C for 6 h	Nanosheet network	[8]
LTO/rGO	Hydrothermal	180 °C for 36 h	600 °C	Spherical	[10]
LTO	Sol–gel	80 °C for 6 h	800 °C for 1 h	Nanosheets	[39]
LTO/carbon nanofibers (5% *w*/*w*)	Sol–gel	60 °C for 24 h	800 °C for 12 h	Urchin-like shape	[9]
LTO/carbon nanofibers	Sol–gel	60°C for 24 h	800 °C for 12 h	Corn-shaped particles	[9]

**Table 3:** Comparison of LTO rate performance with the incorporation of carbon additives.

Anodes	Samples Morphology	Capacity (mAhg^−1^)/Discharge C-Rate	Annealing Temperature (°C)	Electrolyte	Reference
LTO	Spherical	160/0.02	850	1MLiPF_6_EC:PC (1:1)	[33]
LTO	Solid-state reaction	144/2	800	1M LiPF_6_EC:PC:DMC (3:2:5)	[34]
C-LTO/graphene	Network structure	177/0.2	850	1M LiPF_6_EC:DEC (1:1)	[35]
LTO NSs	Spherical	172/0.1	500	1M LiPF_6_EC:DMC (1:1)	[36]
LTO	Nanosheets	175	550	1M LiPF_6_ EC:DEC (1:1)	[37]
LTO	Nanosheets	170	500		[39]
LTO/rGO	Spherical	167/162 after 100 cycles/0.5	600	1M LiPF_6_ EC:DMC (1:1)	[10]
LTO/CNTs	Nanosheet network	145/135 after 1000 cycles/2	700	1M LiPF_6_ EC:DMC:EMC (1:1:1)	[8]
